# A Comparative Analysis of Oral Health and Self-Rated Health: ‘All of Us Research Program’ vs. ‘Health and Retirement Study’

**DOI:** 10.3390/ijerph21091210

**Published:** 2024-09-13

**Authors:** Jane A. Weintraub, Kevin L. Moss, Tracy L. Finlayson, Judith A. Jones, John S. Preisser

**Affiliations:** 1 Department of Pediatric Dentistry and Dental Public Health, Adams School of Dentistry, University of North Carolina, Chapel Hill, NC 27599, USA; 2 Department of Biostatistics and Health Data Science, School of Medicine, Indiana University, Indianapolis, IN 46202, USA; kevmoss@iu.edu; 3 Adams School of Dentistry, University of North Carolina, Chapel Hill, NC 27599, USA; 4 Division of Health Management and Policy, School of Public Health, San Diego State University, San Diego, CA 92182, USA; tfinlays@sdsu.edu; 5 University of Detroit Mercy School of Dentistry, Detroit, MI 48208, USA; jonesja16@udmercy.edu; 6 Department of Biostatistics Gillings, School of Global Public Health, University of North Carolina, Chapel Hill, NC 27599, USA; jpreisse@bios.unc.edu

**Keywords:** oral health, aged, older adults, self-rated health, dental care, edentulous, all of us, electronic health records, surveys, health and retirement study, epidemiology

## Abstract

Poor oral health can impact overall health. This study assessed the association between dental factors (dentate status and dental utilization) and self-rated health (S-RH) among older adults in two cross-sectional datasets: (1) NIH “All of Us (AoU) Research Program” (May 2018—July 2022 release) and (2) U.S. nationally representative “Health and Retirement Study” (HRS) 2018 wave. Participants aged ≥ 51 years were included in these analyses if (1) from AoU, they had clinical dental and medical data from electronic health records (EHRs) and surveys (n = 5480), and (2) from HRS, they had dental and socio-demographic survey data (n = 14,358). S-RH was dichotomized (fair/poor vs. better) and analyzed with logistic regression. Sample survey weights for HRS and stratification and averaging AoU results used the weighted HRS race–ethnicity and age distribution standardized respective analyses to the U.S. population. Fair/poor S-RH was reported by 32.6% in AoU and 28.6% in HRS. Dentate status information was available from 7.7% of AoU EHRs. In population-standardized analyses, lack of dental service use increased odds of fair/poor S-RH in AoU, OR (95% CI) = 1.28 (1.11–1.48), and in HRS = 1.45 (1.09–1.94), as did having diabetes, less education, and ever being a smoker. Having no natural teeth was not statistically associated with fair/poor S-RH. Lack of dental service was positively associated with fair/poor S-RH in both datasets. More and better oral health information in AoU and HRS are needed.

## 1. Introduction

The United States (US) population is aging rapidly. Americans are living longer and experiencing more chronic conditions. Many chronic diseases and poor oral conditions share common risk factors, e.g., poor diet, smoking, stress [1], and loneliness [2]. Biologic mechanisms affecting health, known as the “hallmarks of aging” [3], including chronic inflammation, increasingly demonstrate oral connections [4]. A growing literature shows associations between oral conditions and approximately 60 systemic health conditions [5].

It is time- and cost-intensive to comprehensively assess multiple health conditions clinically. Health data are often collected via self-report using survey questions to quickly assess and monitor population health. A single-item question about self-rated health (S-RH) using a 4- or 5-point scale is frequently used in population-health studies [6,7]. S-RH is a “global” or holistic construct that assesses perceived health status that respondents can base on their different weightings of function, symptoms, diseases, and mental health [8]. S-RH can vary by factors such as age and race/ethnicity [9]. There are gaps in the literature about the linkage between S-RH and oral health among older adults, and limited data sources that include both S-RH and oral health measures for this age group.

Poor oral health, including partial or complete tooth loss, negatively affects oral health-related quality of life [10,11] and functional status [12,13]. Tooth retention is critical for major life activities, e.g., speaking, social interactions, eating and chewing [12,14,15,16,17]. In a longitudinal study of Japanese older adults, declining chewing ability and infrequent routine dental visits were related to adverse health outcomes and mortality [18]. Periodontitis predicted poorer S-RH in another Japanese longitudinal study [19]. Among Ecuadorian older adults, the frequency of reporting fair/poor health increased with increasing number of missing teeth. For example, if missing less than four teeth compared to being edentulous (missing all natural teeth), the adjusted odds ratio (OR) of fair/poor S-RH increased from 1.35 (CI = 0.75–2.43) to OR = 1.88 (1.06–3.32) [20]. Similarly, increasing severity of tooth loss was associated with increasing odds of poor S-RH in studies in Slovenia among 25–64-year-olds [21] and in a Costa Rican retirement cohort [22]. In India, edentulous adults aged 60 years and older had more than twice the odds of reporting fair/poor S-RH compared to dentate adults (OR = 2.38, CI = 1.99, 2.83) [23].

Racial/ethnic disparities exist for tooth loss [24] and dental service use in the US [25]. Edentulous older adults are less likely to have regular dental visits [25,26] and more likely to have chronic health conditions [27]. National cross-sectional data from 2002–2018 show higher crude and adjusted prevalence of dental visits among Whites compared to Black, Hispanic, and minority populations; disparities worsen among older age groups [28].

There are two landmark, independent, large-scale, US National Institutes of Health (NIH)-funded datasets that include both SR-H and oral health information for older adults, the nationally representative, population-based “*Health and Retirement Study*” (HRS) and the ongoing “*All of Us* (AoU) *Research Program*”. The HRS is a biennial, longitudinal study of adults 51 years and older until their death, described as the most “comprehensive population-representative study of aging in the USA” [29]. Initiated in 1992, it is conducted by the University of Michigan and supported by the NIH, National Institute of Aging, and the Social Security Administration. Additional birth cohorts are enrolled periodically, maintaining approximately 20,000 participants at a given time.

AoU is a national longitudinal cohort study originally launched as the NIH Precision Medicine Initiative. Participant enrollment began in 2018 with a goal to gather multiple types of health and related data from one million US participants [30]. One of its primary long-term objectives is to increase the diversity of participants, enrolling people traditionally underrepresented in biomedical research, individuals who identify as other than White and Non-Hispanic, people over 65 years old, individuals with physical or cognitive disability, lower annual household incomes, and less than high school education [31]. Physical measurements, bioassays, and physical activity data are collected for subsets of participants.

The National Institute of Dental and Craniofacial Research (NIDCR) 2021–2026 strategic plan [32] Strategic Priority #1 is to “Integrate Oral and General Health”. One of the goals is “Integration of DOC [dental, oral and craniofacial] conditions with systemic diseases using All of Us data”. Strategic Priority #2 Precision Dental Medicine includes “Addition of dental records into healthcare data ecosystems and infrastructure—such as NIH’s All of Us—to firmly establish the interconnectedness of oral health with overall health”.

In line with the NIDCR’s strategic plan, the primary goal of our study was to evaluate the relationships between S-RH and oral health among older adults, dentate status, and dental care use. By analyzing two large US datasets, we leverage their complimentary aspects to advance our understanding of older adults’ S-RH and oral health. A secondary goal was to explore whether the strength of the relationships between S-RH and oral health among older adults differ between these two studies considering their differing recruitment methods, study populations, and types of data collected. A third goal was to describe the advantages and disadvantages of using the available AoU data for studying oral health and make recommendations for improvement of AoU and HRS. To our knowledge, this analysis is among the first, if not the first, to use AoU survey and electronic health record (EHR) data to study oral health.

## 2. Methods

Study plans for analyzing the HRS and AoU data were reviewed by the University of North Carolina at Chapel Hill Institutional Review Board (IRB) (HRS study IRB #20-2429, 23 August 2020; AoU study IRB #23-008, 31 January 2023). The IRB determined that our studies did not meet the definition of human subjects research under federal regulations; therefore, they did not require IRB approval. No direct contact with study participants occurred during our secondary data analysis of de-identified data. Participants had previously provided informed consent as documented by HRS [33] and AoU [34]. This report adheres to the STROBE (Strengthening the Reporting of Observational Studies in Epidemiology) cross-sectional reporting guidelines, ensuring comprehensive and transparent reporting of our methods and findings [35].

### 2.1. Data Sources

#### 2.1.1. The Health and Retirement Study (HRS) and the All of Us (AoU) Research Program

The HRS provides publicly accessible data online. Core survey data are collected in two-year cycles. We used the cross-sectional self-reported survey data from the 2018 wave, analyzing them with SAS software version 9.4 (SAS Institute, Cary, NC, USA). Survey sampling weights were available. 

While some AoU data snapshots are publicly accessible, the “controlled tier” dataset is only available on a secure, cloud-based researcher workbench for registered users [30]. The researcher workbench is carefully designed to ensure the security and confidentiality of the participants’ information. Data cannot be exported to be analyzed with other software programs and other datasets. In this case, HRS data could not be imported to the workbench to make direct comparisons. At the time, the R software (version 4.4.0 (2024-04-24) was required to conduct analyses. Subsequently, SAS became an option, and our analyses were conducted using SAS version 9.4 (SAS Institute, Cary, NC, USA). We analyzed cross-sectional survey and EHR data collected from May 2018 to the July 2022 data release date [36], the data available at the time of our analysis. The AoU study is broad-scale and geographically distributed but does not use a statistical survey sampling frame for obtaining a nationally representative sample.

#### 2.1.2. Eligibility Criteria

Demographic inclusion criteria were based on age, race/ethnicity, and gender. The HRS only includes adults aged 51 years and older; consequently, we restricted our AoU sample to the same age range. For stratification and weighting purposes, analyses in both datasets were limited to individuals identifying as Non-Hispanic White, Non-Hispanic Black, or Hispanic. Only male and female self-reported genders were included since other categories constituted less than 1% or were unavailable in HRS for comparison.

Exclusion criteria were based on absence of informed consent and data on key variables. Exclusions were applied to prisoners and individuals lacking informed consent (AoU) or legal representative consent. Additional exclusions were based on missing data from the surveys or EHRs (as detailed in Results and in Table 1).

### 2.2. Measurements

In HRS, all variables were determined by survey responses. In AoU, dentate status was determined using EHR data from both AoU procedures and the conditions files. AoU clinical conditions are based on diagnostic/treatment “systematized nomenclature of medicine” (SNOMED) clinical codes in the EHRs. Some individuals had data from multiple healthcare visits. A clinical condition was considered to be present if recorded at any visit during the study time period. If the condition was absent from the patient’s chart, it was assumed that they did not have that condition, which would introduce selection bias if the condition was undiagnosed or unrecorded, potentially under-representing certain conditions and subgroups of individuals. Conversely, conditions, if present, were assumed likely to be included for billing purposes. The AoU program acknowledges that EHR data may be “incomplete and inaccurate” for epidemiologic studies [30].

Outcome variable: S-RH was assessed using similar questions in both HRS and AoU. HRS asked, “Would you say your health is…” while AoU asked, “In general would you say your health is…” Both surveys used the same 5-point response scale dichotomized as Excellent, Very Good, or Good vs. Fair or Poor [37].

Dental measures: Only two types of dental measures are available in the HRS Core data collected from all participants: status and dental visits. In the HRS Core, participants are asked, “In the last two years have you seen a dentist for dental care, including dentures?” AoU includes a similar measure of dental utilization, albeit with a different time frame. In AoU, dental utilization was assessed by the question: “During the past 12 months, have you seen or talked to any of the following doctors or health care providers about your own health?” One of the response options was “A dentist or orthodontist?”.

Dentate status in HRS was determined by the response to the question, “Have you lost all of your upper and lower natural permanent teeth?” Conversely, because AoU does not include a survey question asking about dentition status, determining if a participant had teeth had to be derived indirectly from diagnostic and treatment variables in the EHR. The variable was derived by aggregating all mentions of conditions or procedure codes related to teeth (e.g., dental caries, restorative treatment, gingivitis, or other periodontal conditions) to classify individuals as dentate. Condition codes exist for edentulous, complete absence of teeth due to caries, periodontal disease, etc. Notably, there were no specific condition codes for full-mouth denture repair or full-mouth extractions in the EHR data. If no dental-related codes, conditions, or procedures were recorded, the dentate status variable was missing.

Covariates: In AoU, sociodemographic variables (age, gender, education, race) and behavioral factors (smoking history) were obtained by participant self-reported survey responses. AoU had many gender response options, but only male and female were included to match HRS. Age was dichotomized into two groups (51–64 years, 65 and older); education level as high school or less vs. some college or more. Cigarette smoking was dichotomized as ever or never smoker. While both studies are more heterogeneous and include additional racial and ethnic groups, AoU aims to encompass populations traditionally underrepresented in research and includes a long list of race and ethnicity options. To maintain analytical rigor, we refrained from grouping additional, dissimilar racial/ethnic groups into a generic “other” category due to insufficient sample sizes of other racial/ethnic groups for analysis. Some categories in AoU and HRS with small numbers are masked to protect respondent confidentiality. The HRS primary race categories of White/Caucasian, Black or African American, and ethnicity category Hispanic/Latino were combined into three categories, Non-Hispanic White/Caucasian, Non-Hispanic Black, African American (or African in AoU), and Hispanic/Latino, because these groups had sufficient numbers to match HRS. (Henceforth, these groups will be referred to as Non-Hispanic White, Non-Hispanic Black, and Hispanic).

Medical variables (diabetes, cardiovascular disease (CVD; combined reports of heart disease and stroke)) and cognition-related variables in AoU were based on the diagnostic/treatment codes recorded in the EHR. AoU included a survey question, “Including yourself, who in your family has had memory loss or impairment? Select all that apply”, and respondents could select themselves if applicable. However, most instances of impairment were coded due to a condition noted in the EHR. In HRS, cognitive status was operationalized by using the HRS “cogtot35” total cognition variable, a summary score of cognitive tests administered to participants (e.g., delayed word recall, backwards count), self-reports (e.g., self-rated memory, date naming), or questions answered by their proxy respondents about the participants’ cognition [38,39]. The score was dichotomized at 23 to separate impaired or not impaired cognitive function.

### 2.3. Statistical Analysis

Preliminary analysis involved tabulating counts and describing relative frequencies of socio-demographic, dentate status, dental utilization, medical, and cognition data in the AoU and unweighted HRS datasets; additionally, sample survey weights were applied to obtain a nationally representative set of HRS counts (rounded to the nearest unit) and relative frequencies. Next, frequency distributions of the dichotomized S-RH outcome were compared across subgroups in the assessment of whether the aforementioned factors were associated with S-RH among AoU participants and in the weighted HRS sample. Based on a Bonferroni adjustment for twenty planned tests at the overall 0.05 significance level, *p*-values from Pearson chi-square tests assessed if differences were statistically significant by each factor at the *p* < 0.0025 level (0.0025 = 0.05/20) in the respective datasets.

In separate analyses of the two datasets, multivariable logistic regression modeling identified factors associated with S-RH, including dentate status and dental care via 95% confidence intervals. In HRS, estimated regression coefficients and their standard errors were determined using the nationally representative HRS sample weights as in the univariate and bivariate analyses described above. In contrast, individual level weights were not available in AoU, so a different weighting approach was undertaken. It considered that AoU participants were often recruited in health centers and medical practices, locations where they may be care-seeking. A consequence of the differential design and recruitment goals of the two studies was that the distributions of demographic characteristics differed between AoU and the population-based HRS study. To enhance comparison and interpretation of statistical analysis across studies, a distinct weighting method was applied to AoU as described in the next paragraph.

Therefore, a two-stage analysis procedure was employed to standardize AoU results to the nationally representative HRS study and thereby to the 2018 US population. In the first stage of the AoU analysis, separate unweighted logistic regression models were fitted to each of six age by race/ethnicity groups (strata) where age was dichotomized at age 65 and race–ethnicity groups were Non-Hispanic White, Non-Hispanic Black, and Hispanic. In the second analysis stage of AoU, the stratum-specific logistic regression model regression coefficients and their variances from the first stage were combined (averaged) using stratum-specific weights into a single final set of estimates. The final standard errors of the coefficients were the square roots of their respective weighted-average variances. The stratum-specific weights were determined by summing all weights for HRS participants in the stratum and dividing by the sum of all HRS weights across the six strata. Thus, by applying stratum-specific HRS weights to the AoU logistic regression results for the six demographic groups, the overall (combined) AoU analysis was standardized to the representatively weighted HRS study population.

Statistical power was determined for the study dataset with smaller sample size, reasoning that parallel statistical analyses in the other study dataset would have at least as much power. Thus, for AoU, a two group Pearson chi-square test with a 5% two-sided significance level has 82% power to detect the difference between the dentate group with a proportion of 0.32 having fair/poor S-RH and the edentulous group with a proportion of 0.37 having fair/poor S-RH (odds ratio of 1.25) when the sample sizes are 4595 and 885 (as observed in this study), respectively (a total sample size of 5480). With a 0.25% two-sided significance level based on Bonferroni adjustment, power is only 45%.

## 3. Results

### 3.1. Participants

The flow diagram illustrating participant selection for both the HRS and AoU analyses is presented in Table 1. Each sample size (N) reported in successive rows reflects the cumulative effect of the applied criteria in the current row and rows above it. In our dataset, 61.1% of the AoU cohort were aged 51 years or older compared to all participants (except one person) of the HRS dataset. Within the HRS dataset, missing survey data for key characteristics resulted in the exclusion of 1960 participants, representing 12.0% of the eligible age group, leaving 14,358 remaining in the analytical dataset. Dentate status was determined directly from specific survey questions in HRS and derived indirectly in AoU from EHR codes for conditions on the researcher workbench. This led to only 7.7% of the eligible age group having determinable dentate status data. Among those whose dentate status could be determined, 68.3% were missing information about the last time they talked to a dentist. Of the remaining AoU 6080 participants included in our analysis, 9.9% had missing survey or EHR data for demographic or disease conditions leaving 5480 in our dataset.

**Table 1:** Study flowchart for the All of Us (AoU) and Health and Retirement (HRS) study populations to obtain sample size for analytic datasets (N is what is left after cumulatively applying criteria in the current and higher rows).

Cohort	AoU ^1^ N	HRS N
Entire Cohort	407,333	16,319
Age 51+	248,970	16,318
Dentate Status (dentate or edentulous)	19,206	16,290
Dentist visit	6080	16,178
Self-Rated Health	6075	16,173
Race: Non-Hispanic Black, Non-Hispanic White Ethnicity: Hispanic	5669	15,282
Sex at Birth, Female or Male	5618	15,282
Education Level	5561	15,282
Smoking (ever or never)	5480	15,282
Cognition ^1^	5480	14,691
Diabetes ^1^	5480	14,504
CHD/Stroke ^1^	5480	14,358

^1^ For AoU: Based on Diagnostic/Treatment Coding (if no code is present, the condition is assumed “Absent”). Other AoU variables and all HRS variables were based on questionnaire self-report or cognitive tests.

### 3.2. Descriptive Characteristics of Participants

In HRS, Hispanic and Non-Hispanic Black population groups are oversampled to increase their sample sizes for analysis. We recalculated relative frequencies using the survey sampling weights to produce counts whose relative frequency distribution (i.e., percentages) represents the national US population at the time the survey was drawn. Table 2 shows the unweighted and population-weighted percentages of the HRS sample in each of the six age-race/ethnicity strata. As expected, the percentages of Non-Hispanic Blacks and Hispanics among HRS participants (i.e., the unweighted sample) are greater than the population-weighted, nationally representative HRS percentages. The latter, when expressed as proportions, are the weights used for combining the stratum-specific AoU logistic regression coefficients with the aim to make both HRS and AoU analyses nationally representative and comparable. Interestingly, the AoU and unweighted HRS sample age–race/ethnicity distributions are similar.

Table 3 presents the observed frequency distributions of the demographic and clinical characteristics and S-RH outcome among AoU and (unweighted) HRS study participants. In both datasets, a majority of the study participants were at least 65 years old, 57% and 55% in AoU and HRS, respectively. The mean (SD) age was 66.9 years (SD = 9.0) for AoU participants and 66.3 (SD = 9.7), for HRS participants.

Overall, the racial and ethnic composition of the AoU and unweighted HRS study populations were more diverse than the weighted HRS population which reflects the 2018 U.S. population, with 18.4% of participants in AoU and 22.7% in HRS identifying as Non-Hispanic Black and 17.0% and 16.4%, respectively, as Hispanic, compared to 10.9% and 10.5% respectively in the weighted HRS. 

Comparing the AoU and unweighted HRS sample characteristics, there are many similarities. The majority of both groups were female, in the older age category, never smokers, and without diabetes, CVD, or cognitive impairment, and S-RH was excellent, very good, or good. Notably, 68–70% had at least some college education. People in the AoU group were more likely to be smokers and in the HRS group more likely to have cardiovascular disease. In both groups, 16% were edentulous despite very different methods of obtaining this information. Dental use was similar though the survey questions were worded differently and reflected different durations of time.

The unweighted HRS sample was not very different from the nationally representative weighted HRS sample, other than the racial and ethnic composition. HRS participants, as indicated by unweighted percentages, were slightly more likely (by 5% to 7% percentage points) to be in the older (65+) age group, to have fair/poor S-RH, to have no recent dentist contact, and to have some college than the HRS weighted sample representing the U.S. population.

### 3.3. Self-Rated Health

Table 4 summarizes characteristics associated with dichotomized S-RH among AoU and weighted HRS participants. In this unadjusted bivariate analysis, all observed differences had *p* ≤ 0.0025 except dentate status in AoU only (*p* = 0.004), age in HRS only (*p* = 0.01), and gender in both studies (AoU *p* = 0.23, HRS *p* = 0.19). In these exceptions, the difference between subgroups in the percentage having fair/poor S-RH was less than 5%. Factors statistically associated with better S-RH in AoU and HRS with all subgroup differences being greater than 8% included being White, older (AoU only), with more recent dentist contact, dentate (HRS only), more education, never smokers, and without diabetes, CVD, or impaired cognition.

Logistic regression analyses (Table 5), weighted to standardize results to the US population (using different methods across the two studies), showed consistency in factors associated with fair/poor S-RH in AoU and HRS models. Adjusted odds ratios indicate increased risk for fair/poor S-RH among those without recent dentist contact, OR = 1.28 in AoU and 1.45 in HRS. In both studies, people with diabetes had more than twice the odds of having unfavorable S-RH compared to those without diabetes. Ever smokers had 1.38 (AoU) and 1.89 (HRS) times higher odds to have unfavorable S-RH compared to never smokers. Having less education statistically significantly increased the odds of fair/poor S-RH in both studies. The HRS model showed modestly higher odds for fair/poor S-RH among people with each of these characteristics compared to odds for AoU except odds of having diabetes was slightly higher for AoU. Being edentulous did not statistically increase the odds of unfavorable S-RH in either study.

Table A1a,b in the Appendix A present results of unweighted logistic regression for the six race/ethnicity by age strata in AoU. It is not surprising that the results in the overall AoU analysis in Table 5 are most similar to results for the two Non-Hispanic White strata since the combining of the six strata to produce the overall model results give 78% weight to Whites as reflecting the US national population. There was only a modest degree of heterogeneity of estimated odds ratios across the six strata (taking into consideration increased uncertainty in estimates for Non-White groups due to smaller sample sizes) such that analytic approach of averaging results across the six logistic regressions to obtain the results in Table 5 was supported. The majority of the six strata reported 95% confidence intervals for less education, ever smoker and diabetes that had lower confidence bounds for adjusted odds ratios greater than 1.0 indicating statistical associations. The estimated odds ratio for the association of no recent dentist contact with fair/poor S-RH was statistically significant (in the sense of the CI for the odds ratio not containing the value of 1.0) only for the two Non-Hispanic White age groups.

## 4. Discussion

After standardizing both datasets to the 2018 US population, lack of dental care, but not dentate status, increased the odds of unfavorable S-RH in both studies. The finding of the association between low dental attendance and fair/poor S-RH aligns with previous research by Manski and colleagues [40]. They found lower dental attendance was associated with fair/poor self-reported health across European countries using data from the Survey of Health, Ageing and Retirement in Europe and in the US from 2004–2006 HRS data. A study by Meyerhoefer and colleagues [27], using a longitudinal 1992–2016 RAND subset of HRS data, also found that visiting a dentist in the past two years is associated with improved S-RH.

Many factors contribute to dental service use, including dentition status. People who are edentulous or with fewer teeth present tend to visit dentists less frequently. Ghanbari-Jahromi and colleagues, in their scoping review of dental services utilization [41], used health-level, access, demographic, social, and financial factors to categorize their findings. Number of teeth was the most cited health-related factor in their review. Reda and colleagues [42], in their systematic review from 28 countries, also found dental utilization was lower for edentulous individuals, among other factors. Conversely, in 6-year [26] and 12-year [43] longitudinal HRS models, poor dental attendance and smoking increased the odds of, or prediction of becoming edentulous. Smoking and diabetes were significantly associated with S-RH in our analysis. Both conditions are risk factors for periodontal disease [44], which, when severe, can lead to tooth loss [45]. Longitudinal analysis possible with HRS data is not yet available with AoU. Developing procedures to make future AoU longitudinal analyses possible will strengthen future research findings.

There are some potential explanations for the lack of a significant association found between dentate status and S-RH in our analyses. Dentate status was determined differently in the two studies. In AoU, it was difficult to determine dentate status because of the magnitude of missing oral health information in the EHRs. This gap limited statistical power for dentate status. If any misclassification of dentition status in AoU occurred because of lack of documentation in the EHR, it may have also contributed to the lack of significant association in the analysis. However, we only assigned non-missing dentate status when dental-related codes were available. With self-reports, misclassification could occur, for example, if people with implant-supported dentures do not consider themselves edentulous. S-RH may also differ among people missing all natural teeth depending on whether they have a satisfactory denture replacement.

We included several health conditions as covariates that have been associated with poor oral health and/or tooth loss. For example, several systematic reviews have explored the relationship between tooth loss and cognitive impairment and dementia, yielding varying results [46,47,48,49,50]. Cohort studies suggest that tooth loss increases the risk of cognitive decline and function in older adults [46] with a dose–response effect indicating increased risk with more teeth lost [47]. This relationship also can be bidirectional. Using 12-year longitudinal data from the US HRS, predictive models incorporating cognitive impairment with other demographic factors and last dental visit improved the prediction of older adults becoming edentulous [43]. In another HRS-based cohort study, older adults with both edentulism and diabetes at the outset experienced worsened and accelerated cognitive decline [51].

### 4.1. Study Strengths

The oral health of older adults in the US is under-studied due in part to the few national large-scale studies that include both clinically determined medical and dental data and other relevant factors assessed by self-report. Recruitment of older populations for research is also challenging. The availability of two large-scale national population studies that include large sample sizes of older adults and extensive survey data about a broad range of health and health care topics, including S-RH, a key gestalt indicator of perceived overall health and dental utilization, was an opportunity to help overcome these challenges. Although the studies have different recruitment methods and study designs, we were able to address this issue with statistical weighting methods.

### 4.2. Limitations and Recommendations for Improvement

#### 4.2.1. Timing and Geography

The HRS data used were collected pre-COVID-19, and the AoU data covers the recruitment period of May 2018 to July 2022, overlapping with the COVID-19 pandemic. Many dental offices were closed, and dental visits declined during the early phases of the pandemic, decreasing access to dental care and affecting the use of dental services [52]. Nevertheless, the percentages of people in our datasets responding positively to the dental utilization question were 64% and 69%.

Based on the AoU website’s US map as of 23 July 2024 of “participants in each state who had completed the initial steps of the program” [53], AoU has some geographic selection bias. The states California and Arizona were overrepresented; each had over 72,000 participants, while 18 states had fewer than 2000 participants. Our results are applicable for participants enrolled up until the data release date at the time of our analyses. Analyses conducted in the future with a larger sample size and more diversity in geography and enrollment settings may yield different results and permit the use of zip codes to add geographic contextual data for studying the impact of social determinants on the health of older adults.

#### 4.2.2. Information from Electronic Health Records

Various challenges emerged when analyzing this AoU clinical data for health services research. Clinical oral health information was very limited, as non-dental health care settings do not routinely collect it unless the clinic shares the same platform (i.e., Epic Systems software) in hospital or other settings with a dental department or the dental condition is symptomatic or relevant to other medical treatment. If a condition was absent in the AoU EHR, assuming that the condition does not exist can lead to an underestimation of its prevalence. For example, assuming that someone did not have periodontal disease, if it was missing as a condition, and there was no procedure listed to treat it, would result in an underestimation of periodontal disease prevalence if present.

This lack of documentation applies to many other oral conditions. For example, it was not possible to determine the extent of tooth loss or if patients were partially edentulous with a denture or missing all teeth in one but not both arches. The quality and perceived satisfaction with any dentures and implants worn was unknown but could certainly affect chewing ability, quality of life, and S-RH [54,55]. There is a need for patient intake and medical history forms to include oral conditions as part of overall health [56]. Medical personnel and other health care providers need to observe and document oral conditions in the EHR. Simon and colleagues investigated the value of sharing medical and dental information among physicians and dentists; both groups agreed that sharing information about oral and systemic health with inter-operable EHRs would benefit coordinated and collaborative patient care [57].

Recruiting AoU participants from private and public dental practice settings is not occurring. Including these locations would aid collection of oral health information, especially from federally qualified health centers (FQHCs) and other settings that include dental clinics and interoperable, shared EHRs. AoU has recognized FQHCs as important sites for recruitment because they traditionally serve populations underrepresented in biomedical research [58]. Many FQHCs serve dental patients [59]. In 2014, 76% of FQHCs nationally provided oral health services [60].

#### 4.2.3. Survey Questions

There were very few oral health-related questions in the AoU online surveys. The “Personal Medical History” survey component included 465 questions without any explicit mention of oral health or dental care. The personal and family history survey was 101 pages and did not include any oral health questions except head and neck cancer. The time since last health care provider visit (including dentist) was included in the “Healthcare access and utilization” survey. The question used was not a standardized question used in other national surveys. Oral health questions need to be added to the AoU and biennial core HRS surveys, including those asked in other national studies such as the National Health and Nutrition Examination Survey (NHANES) that have been validated [61]. HRS has added oral health questions via “experimental modules” in their 2008 and 2018 waves, each with about a 10% subsample of the CORE participants [62]. As with all surveys, methods to increase response rates are desirable.

## 5. Conclusions and Recommendations

The AoU Research Program brings together an extraordinary array of information to understand disease and improve health. We standardized the data to the US population and compared it to the nationally representative US HRS to examine the association of S-RH with oral health factors. Among adults over age 50, lack of contact with a dentist in the past one or two years, increased the odds of fair or poor S-RH, as did ever smoking, having diabetes, and less education, but not whether they had any natural teeth. The direct relationship between use of dental services and perceived health provides evidence that obtaining oral health care should be included in comprehensive health promotion activities.

Recommendations are made to improve the utility of AoU and HRS for epidemiologic and health services research. These include adding oral health-related, standardized survey questions, improving documentation of oral health information in EHRs, and recruiting AoU participants from public and private dental practices. Improving the AoU and HRS studies for future oral health research could advance our understanding of the interconnectedness and integration of oral health and primary care for older adults.

## Figures and Tables

**Table 2:** Distribution of age group by race/ethnicity in Health and Retirement Study (HRS) and All of Us (AoU) Research Program.

Race/Ethnicity	AoU (N = 5480)		HRS Unweighted (N = 14,358)		HRS Weighted ^1^ (N = 14,358)	
	51–64	65+	51–64	65+	51–64	65+
Non-Hispanic Black	568 (10.3%)	442 (8.1%)	1847 (12.9%)	1414 (9.9%)	894 (6.2%)	675 (4.7%)
Non-Hispanic White	1297 (23.7%)	2241 (40.9%)	3203 (22.3%)	5535 (38.6%)	5301 (36.9%)	5975 (41.6%)
Hispanic	469 (8.6%)	463 (8.5%)	1357 (9.5%)	1002 (7.0%)	905 (6.3%)	608 (4.2%)
All Race/Ethnicity Groups	2334 (42.6%)	3146 (57.4%)	6407 (44.6%)	7951 (55.4%)	7100 (49.5%)	7258 (50.5%)

^1^ The HRS weighted relative frequencies are the stratification weights (as proportions) in the multivariable AoU analysis; the counts are the reweighted counts, rounded to the nearest unit. Cell frequencies are shown for the six race/ethnicity by age groups. The last row shows marginal relative frequencies for all race/ethnicity groups combined.

**Table 3:** Demographic, dental, and medical characteristics of AoU (2018–2022) and HRS 2018 participants above age 50 years.

Variables	AoU (Column %)	HRS Unweighted (Column %)	HRS Weighted (Column % ^1^)
Race/Ethnicity Non-Hispanic Black Non-Hispanic White Hispanic	1010 (18.4%) 3538 (64.6%) 932 (17.0%)	3261 (22.7%) 8738 (60.9%) 2359 (16.4%)	1569 (10.9%) 11,275 (78.5%) 1512 (10.5%)
Sex Female Male	3385 (61.8%) 2095 (38.2%)	8416 (58.6%) 5942 (41.4%)	7783 (54.2%) 6574 (45.8%)
Age (years) 51–64 65+	2334 (42.6%) 3146 (57.4%)	6407 (44.6%) 7951 (55.4%)	7100 (49.5%) 7257 (50.6%)
Self-Rated Health Excellent/Very Good/Good Fair/Poor	3696 (67.5%) 1784 (32.6%)	10,255 (71.4%) 4103 (28.6%)	10,967 (76.4%) 3390 (23.6%)
Recent Dentist Contact Yes No	3793 (69.2%) 1687 (30.8%)	9156 (63.8%) 5202 (36.2%)	9937 (69.2%) 4420 (30.8%)
Dentate Status Edentulous Dentate	885 (16.1%) 4595 (83.9%)	2300 (16.0%) 12,059 (84.0%)	1845 (12.9%) 12,511 (87.2%)
Education HS or Less Some College or More	1632 (29.8%) 3848 (70.2%)	4569 (31.8%) 9789 (68.2%)	5573 (38.8%) 8784 (61.2%)
Ever Smoker Yes No	2577 (47.0%) 2903 (53.0%)	4825 (33.6%) 9533 (66.4%)	4870 (33.9%) 9487 (66.1%)
Diabetes Yes No	1105 (20.2%) 4375 (79.8%)	3989 (27.8%) 10,369 (72.1%)	3435 (23.9%) 10,922 (76.1%)
CVD Yes No	223 (4.1%) 5257 (95.9%)	4007 (27.9%) 10,351 (72.1%)	3681 (25.6%) 10,676 (74.4%)
Impaired Cognition Yes No	279 (5.1%) 5201 (94.9%)	704 (4.9%) 13,654 (95.1%)	969 (6.8%) 13,388 (93.3%)

^1^ Weighted to US population. Variables are collected differently for each study; refer to methods.

**Table 4:** Study variables by dichotomized self-rated health among AoU (2018–2022) and HRS 2018 participants in analytic dataset.

	AoU			Weighted HRS		
	Excellent Very Good Good N = 3269	Fair Poor N = 1832	*p*-Value *	Excellent Very Good Good N * = 10,967	Fair Poor N * = 3390	*p*-Value *
Race/Ethnicity Non-Hispanic Black Non-Hispanic White Hispanic	570 (56.4%) 2616 (73.9%) 510 (54.7%)	440 (43.6%) 992 (26.1%) 422 (45.3%)	<0.001	1039 (66.2%) 9044 (80.2%) 884 (58.5%)	530 (33.8%) 2232 (19.8%) 628 (41.6%)	<0.001
Sex Female Male	2263 (66.9%) 1433 (68.4%)	1122 (33.2%) 662 (31.6%)	0.23	5912 (76.0%) 5054 (76.9%)	1871 (24.0%) 1519 (23.1%)	0.19
Age (years) 51–64 65+	1435 (61.5%) 2261 (71.9%)	899 (38.5%) 885 (28.1%)	<0.001	5487 (77.3%) 5480 (75.5%)	1613 (22.7%) 1777 (24.5%)	0.01
Recent Dentist Contact Yes No	2743 (72.3%) 953 (56.5%)	1050 (27.7%) 734 (43.5%)	<0.001	8207 (82.6%) 2760 (62.4%)	1730 (17.4%) 1661 (37.6%)	<0.001
Dentate Status Edentulous Dentate	560 (63.3%) 3136 (68.3%)	325 (36.7%) 1459 (31.8%)	0.004	1083 (58.7%) 9883 (79.0%)	762 (41.3%) 2628 (21.0%)	<0.001
Education HS or Less ** Some College ***	886 (54.3%) 2810 (73.0%)	746 (45.7%) 1038 (27.0%)	<0.001	6132 (69.8%) 4835 (86.8%)	2652 (30.2%) 738 (13.2%)	<0.001
Ever Smoker Yes No	1588 (61.6%) 2108 (72.6%)	989 (38.4%) 795 (27.4%)	<0.001	3384 (69.5%) 7582 (79.9%)	1486 (30.5%) 1904 (20.1%)	<0.001
Diabetes Yes No	544 (49.2%) 3152 (72.1%)	561 (50.8%) 1223 (28.0%)	<0.001	2104 (61.3%) 8862 (81.1%)	1331 (38.7%) 2060 (18.9%)	<0.001
CVD Yes No	126 (56.5%) 3570 (67.9%)	97 (43.5%) 1687 (32.1%)	<0.001	2256 (61.3%) 8711 (81.6%)	1426 (38.7%) 1964 (18.4%)	<0.001
Impaired Cognition Yes No	166 (59.5%) 3530 (67.9%)	113 (40.5%) 1671 (32.1%)	<0.001	872 (90.0%) 10,095 (75.4%)	97 (10.0%) 3294 (24.6%)	<0.001

* Pearson chi-square test; ** High School or Less; *** Some College or More.

**Table 5:** Adjusted odds ratio and 95% confidence intervals for fair or poor self-rated health by study standardized to 2018 US population ^1^.

Characteristic	AoU Fair/Poor S-RH OR (95% CI)	HRS Fair/Poor S-RH OR (95% CI)
Male	0.96 (0.83–1.10)	0.94 (0.70–1.25)
No Recent Dentist Contact	1.28 (1.11–1.48)	1.45 (1.09–1.94)
Edentulous	1.14 (0.78–1.65)	1.57 (0.69–3.57)
Less Education	1.24 (1.06–1.45)	1.42 (1.03–1.94)
Ever Smoker	1.38 (1.20–1.59)	1.89 (1.43–2.51)
Diabetes	2.33 (1.68–3.22)	2.23 (1.60–3.09)
Cardiovascular disease	1.72 (0.80–3.72)	1.33 (0.65–2.75)
Impaired Cognition	1.97 (0.94–4.16)	1.99 (0.87–4.59)

^1^ Results are based on logistic regression applied to each study. For HRS, we used nationally representative, sample survey weights for study participants. For AoU, estimates from six stratum-specific unweighted logistic regression models were combined based upon the weighted distribution of age (<65, 65+) and race/ethnicity groups in HRS.

## Data Availability

Publicly available data used in this analysis are online at the Health and Retirement Study website: https://hrs.isr.umich.edu/data-products (accessed on 11 May 2021). The All of Us data are publicly available online for the “public tier” and the “registered tier” for approved researchers to access individual-level data from surveys and EHRs [Data Access Tiers–All of Us Research Hub (https://researchallofus.org/data-tools/workbench/ (accessed on 25 July 2024))].

## Appendix A

**Table A1:** Results from All of Us logistic regression models for race/ethnicity and age strata.

**a. Regression Coeficients (Standard Errors)**						
	**Non-Hispanic Black** **51–64** **N = 568**	**Non-Hispanic Black** **65+** **N = 442**	**Non-Hispanic** **White** **51–64** **N = 1297**	**Non-Hispanic White** **65+** **N = 2241**	**Hispanic** **51–64** **N = 469**	**Hispanic** **65+** **N = 463**
Male	0.03 (0.09)	−0.06 (0.11)	−0.10 (0.07)	0.00 (0.05)	0.07 (0.12)	−0.29 (0.11)
No Recent Dentist Contact	0.13 (0.09)	0.16 (0.10)	0.36 (0.07)	0.22 (0.06)	0.16 (0.10)	−0.08 (0.10)
Edentulous	−0.38 (0.23)	0.07 (0.23)	0.24 (0.20)	0.14 (0.14)	−0.02 (0.26)	−0.04 (0.22)
Less Education	0.08 (0.09)	0.30 (0.10)	0.28 (0.08)	0.15 (0.07)	0.27 (0.10)	0.23 (0.11)
Ever Smoker	0.23 (0.09)	0.20 (0.11)	0.38 (0.06)	0.32 (0.06)	0.20 (0.11)	0.35 (0.11)
Diabetes	0.61 (0.20)	0.92 (0.22)	0.88 (0.17)	0.87 (0.13)	0.81 (0.22)	0.54 (0.21)
Cardiovascular Disease	0.88 (0.56)	0.91 (0.47)	0.95 (0.39)	0.20 (0.21)	−0.08 (0.59)	0.42 (0.79)
Impaired Cognition	1.22 (0.60)	0.69 (0.53)	0.93 (0.30)	0.35 (0.18)	0.19 (0.70)	1.67 (0.84)
**b. Odds Ratio (95% Confidence Interval)**						
	**Non-Hispanic Black** **51–64** **(N = 568)**	**Non-Hispanic Black** **65+** **(N = 442)**	**Non-Hispanic** **White** **51–64** **(N = 1297)**	**Non-Hispanic White** **65+** **(N = 2241)**	**Hispanic** **51–64** **(N = 469)**	**Hispanic** **65+** **(N = 463)**
Male	1.07 (0.75–1.53)	0.89 (0.58–1.37)	0.82 (0.63–1.07)	1.00 (0.81–1.24)	1.14 (0.73–1.80)	0.56 (0.36–0.88)
No Recent Dentist Contact	1.32 (0.92–1.85)	1.39 (0.93–2.08)	2.06 (1.58–2.69)	1.56 (1.23–1.98)	1.39 (0.94–2.04)	0.85 (0.57–1.27)
Edentulous	0.68 (0.43–1.08)	1.08 (0.68–1.70)	1.28 (0.86–1.90)	1.15 (0.87–1.53)	0.98 (0.59–1.63)	0.96 (0.63–1.47)
Less Education	1.19 (0.83–1.69)	1.81 (1.20–2.71)	1.75 (1.30–2.36)	1.35 (1.03–1.78)	1.73 (1.16–2.58)	1.58 (1.04–2.40)
Ever Smoker	1.59 (1.12–2.26)	1.49 (0.98–2.25)	2.15 (1.67–2.77)	1.88 (1.52–2.34)	1.49 (0.97–2.29)	2.02 (1.30–3.13)
Diabetes	1.86 (1.26–2.72)	2.50 (1.63–3.84)	2.42 (1.72–3.41)	2.39 (1.86–3.07)	2.25 (1.47–3.46)	1.72 (1.13–2.61)
Cardiovascular Disease	2.42 (0.80–7.29)	2.48 (0.98–6.27)	2.59 (1.20–5.62)	1.22 (0.80–1.84)	0.92 (0.29–2.91)	1.52 (0.33–7.08)
Impaired Cognition	3.40 (1.04–11.0)	2.00 (0.70–5.69)	2.53 (1.41–4.56)	1.42 (0.99–2.03)	1.21 (0.30–4.82)	5.33 (1.02–27.9)
